# The Burden of Progressive-Fibrosing Interstitial Lung Diseases

**DOI:** 10.3389/fmed.2022.799912

**Published:** 2022-02-01

**Authors:** Vincent Cottin, Rhiannon Teague, Lindsay Nicholson, Sue Langham, Mike Baldwin

**Affiliations:** ^1^Louis Pradel Hospital, Reference Center for Rare Pulmonary Diseases, Hospices Civils de Lyon, Lyon, France; ^2^Claude Bernard University Lyon 1, UMR754, IVPC, Member of OrphaLung, RespiFil, Radico-ILD and ERN-LUNG, Lyon, France; ^3^Maverex Limited, Manchester, United Kingdom; ^4^Boehringer Ingelheim International GmbH, Ingelheim am Rhein, Germany

**Keywords:** progressive fibrosing ILD, epidemiology, survival, humanistic burden, quality of life, economic burden

## Abstract

Despite conventional treatment, a proportion of interstitial lung disease (ILD) patients develop a progressive phenotype known as “fibrosing ILD with a progressive phenotype” (PF-ILD), characterized by worsening respiratory symptoms, decline in lung function, and early mortality. This review describes the epidemiology, and the humanistic and economic burden of PF-ILDs other than idiopathic pulmonary fibrosis (non-IPF PF-ILD). A structured review of the literature was conducted, using predefined search strategies in Ovid MEDLINE and EMBASE, and supplemented with gray literature searches. The search identified 3,002 unique articles and an additional 3 sources were included from the gray literature; 21 publications were included. The estimated prevalence of non-IPF PF-ILD ranges from 6.9 to 70.3/100,000 persons and the estimated incidence from 2.1 to 32.6/100,000 person-years. Limited evidence demonstrates that PF-ILD has a significant impact on patients' quality of life, affecting their daily lives, psychological well-being, careers, and relationships. PF-ILD is also associated with significant economic burden, demonstrating higher healthcare resource use and direct costs compared with the non-progressive phenotype, and indirect costs, which include job losses. This review indicates that PF-ILD places a considerable humanistic burden on both patients and caregivers, and a substantial economic burden on healthcare systems, patients, and society.

## Introduction

Interstitial lung diseases (ILDs), also referred to as diffuse parenchymal lung diseases, encompass a large and diverse group of restrictive lung conditions, overlapping in their clinical presentations and patterns of lung injury (1). Despite conventional treatment, a significant proportion of patients with certain types of ILDs will develop a progressive phenotype comparable to untreated idiopathic pulmonary fibrosis (IPF), characterized by worsening respiratory symptoms, decline in lung function, and early mortality (2, 3). Although not every patient develops a progressive phenotype, those that do exhibit a similar disease course and prognosis to patients with IPF (2, 4). Consequently, ILDs exhibiting this progressive phenotype are grouped with IPF under the term “fibrosing ILD with a progressive phenotype” (PF-ILD) (5), also designated progressive pulmonary fibrosis (4).

ILDs most likely to develop a progressive phenotype are idiopathic non-specific interstitial pneumonia (iNSIP), unclassifiable ILD (u-ILD), fibrotic hypersensitivity pneumonitis (HP) and ILDs associated with autoimmune disorders, particularly rheumatoid arthritis-associated ILD (RA-ILD) and systemic sclerosis-associated ILD (SSc-ILD) (6, 7). Currently, no formal criteria to assess disease progression in patients with ILDs exists. In clinical trials and in practice, progression is typically evaluated through the serial assessment of lung function, together with symptoms and imaging features (1, 7, 8).

Clinical events such as acute exacerbations (AEs) and respiratory hospitalizations indicate disease worsening (1, 7). Common and burdensome symptoms of PF-ILD include dyspnea, cough and fatigue (9). AEs of PF-ILD are clinically-meaningful events characterized by rapid respiratory deterioration (10), associated with a poor prognosis and increased mortality (11).

In addition to the absence of formal criteria to assess disease progression in clinical practice, there is no available International Classification of Diseases (ICD) coding outside of the US [ICD-10-Clinical Modification (CM) J84.170 introduced 2020] to identify ILD patients exhibiting this progressive phenotype in real-world datasets (6, 12). Consequently, this leads to inconsistent approaches used to identify PF-ILD patients in the real world, including clinical trial criteria, algorithms, and expert consensus (3, 6, 11).

The pathogenesis of PF-ILD is not fully understood, although certain pro-fibrotic cellular and molecular mechanisms have been established as a common feature of progressive self-sustaining pulmonary fibrosis (13). The assumed shared pathobiological mechanisms across PF-ILDs indicate that disease progression may be slowed down in response to similar types of treatment, targeting the underlying fibrosis (5). Until recently, no medicines were approved for the treatment of any PF-ILDs other than IPF (7). Since March 2020, the antifibrotic, nintedanib, has received marketing authorization in several regions including Europe (14), the US (15), Canada (16), and Japan (17), based on consistent slowing in progression of lung fibrosis in a broad range of PF-ILDs in the INBUILD trial (8).

Although there is a good understanding of the epidemiology and burden of IPF, the archetypal and most studied PF-ILD (18–20), little is known about the burden of other PF-ILDs as a group. The objective of this review is to identify and synthesize studies on the epidemiology and the humanistic and economic impact of PF-ILDs other than IPF (non-IPF PF-ILDs).

## Materials and Methods

Searches for full-text reports containing original data were run in Ovid MEDLINE and EMBASE in March 2021 and an update conducted on the 14th June 2021. Thesaurus terms (MeSH and Emtree for MEDLINE and Embase, respectively), and subject headings were combined with free-text keywords. The detailed search strategy is available in Supplementary Table 1.

Reference lists of articles were searched and handsearching of conference abstracts was performed for the final 2 years of the search period (2020–2021). We included full publications of studies published in English reporting on adults with non-IPF PF-ILDs and any of the following variables: epidemiology (including prevalence, incidence, proportion of fibrosing ILDs with a progressive phenotype, survival); humanistic burden [including patient/carer HRQoL and patient-reported outcome or experience measures (PROMs/PREMs)]; and economic burden [including healthcare resource use (HCRU), healthcare costs, and productivity losses].

## Results

### Literature Search Results

**Figure 1:**
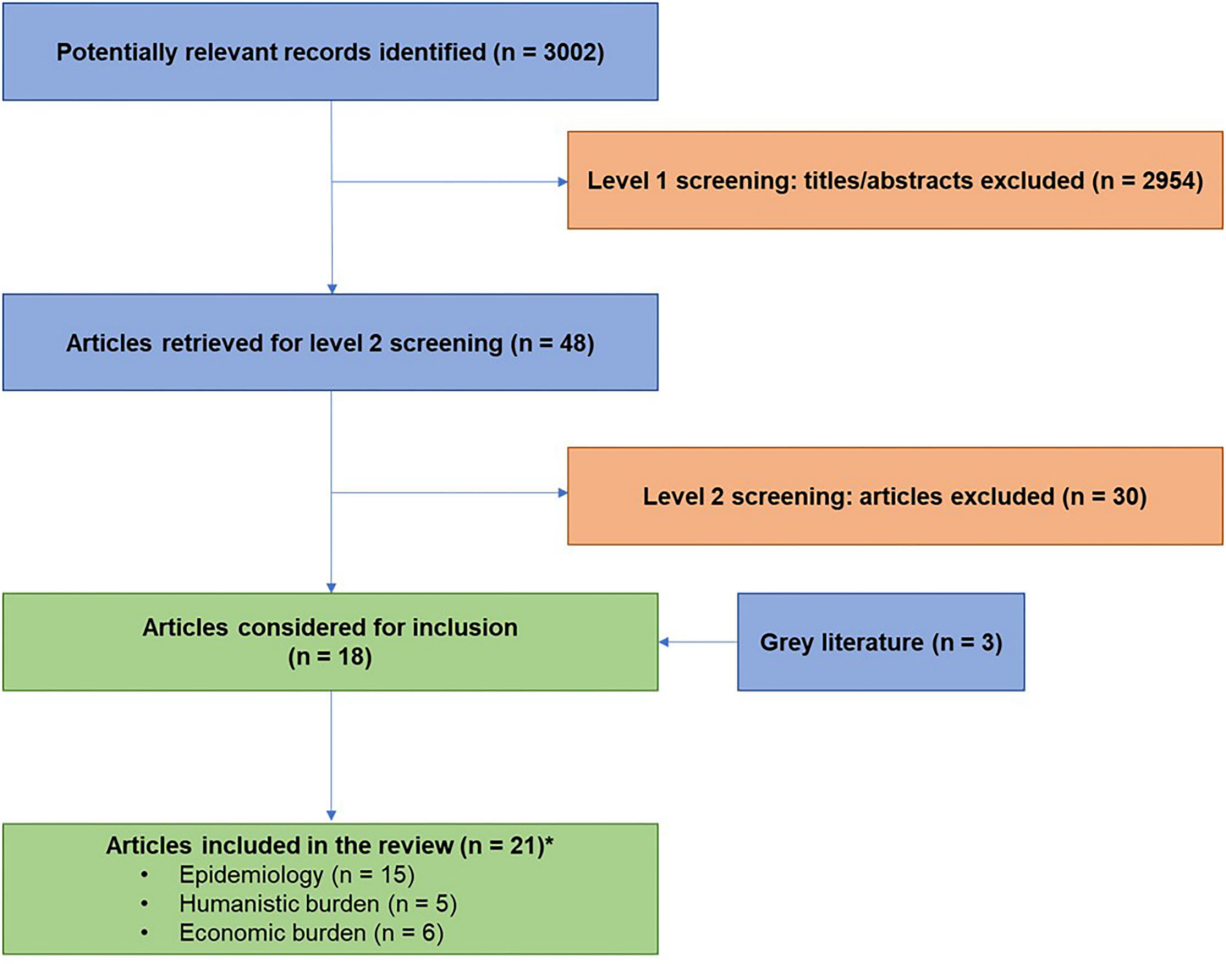
Flow diagram of study inclusion. *Some articles overlapped in their datasets.

The search identified 3,002 unique articles. At title and abstract screening and then full text screening 2,954 and 30 were excluded, respectively. In total, 18 were included in the review. In addition, 3 sources were included from the gray literature. The review included 21 publications that reported on epidemiology (*n* = 15), humanistic burden (*n* = 5), and economic burden (*n* = 6) (Figure 1).

### Epidemiology

#### Prevalence and Incidence

**Figure 2:**
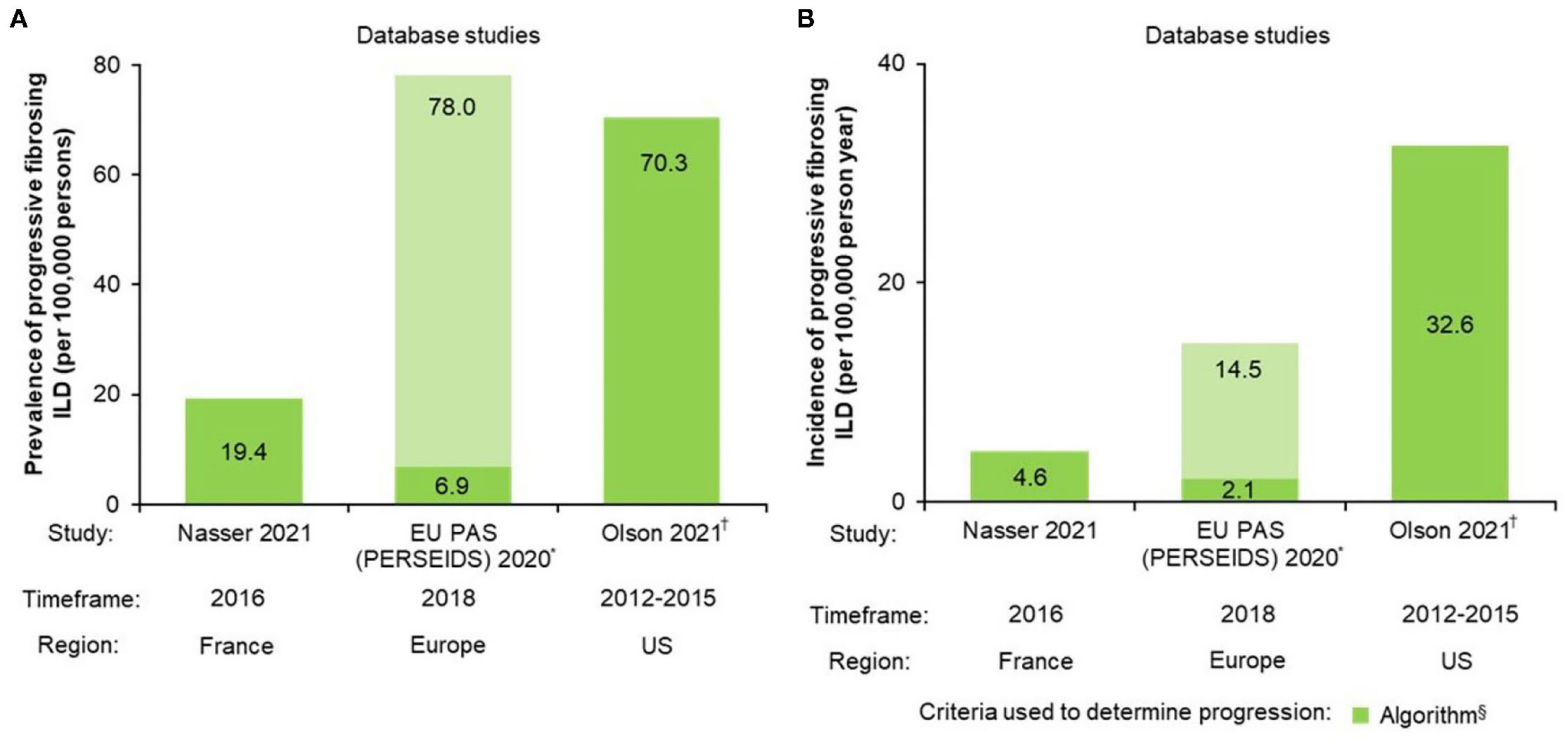
Non-IPF PF-ILD **(A)** prevalence and **(B)** incidence. *Estimates vary by country; the lower number represents the minimum PPV adjusted value and the higher number the crude maximum value. PPV is based on whether fibrosing ILDs were actually fibrosing ILDs; ^†^Age- and sex-adjusted (standardized to the 2014 US Census estimates) ^§^Algorithms for definition of progression were specifically designed for each study; see Table 1 for further algorithm details. EU, European Union; PAS, post-authorization study; PF-ILD, progressive fibrosing interstitial lung disease; PPV, positive predictive value.

Three data sources reported prevalence and/or incidence estimates for patients with PF-ILDs (Table 1, Figure 2). Estimated prevalence of non-IPF PF-ILD ranged from 6.9 (Europe) to 70.3 (US)/100,000 persons and estimated incidence ranged from 2.1 (Europe) to 32.6 (US)/100,000 per person-years.

**Table 1:** Studies reporting proportion of non-IPF fibrosing ILDs with progressive phenotype, incidence and prevalence of progressive fibrosing ILD.

**References, country**	**Study design, no. of non-IPF fibrosing ILD patients**	**Study years**	**Criteria used to determine progression**	**Proportion of fibrosing ILDs with progressive phenotype (*n* progressive phenotype/*n* fibrosing ILD other than IPF)**	**Incidence per 100,000 person-years**	**Prevalence per 100,000 persons**
Nasser et al. (21), France	Clinical cohort (*n* = 617)	Jan 2010–Dec 2017	INBUILD criteria^*^	27.2% (168/617)	NR	NR
Simpson et al. (22), UK	Clinical cohort (*n* = 1,749)	Aug 2017–Jan 2018	INBUILD criteria^*^	14.5% (253/1,749)	NR	NR
Faverio et al. (23), Italy	Clinical cohort (*n* = 245)	Jan 2011–Jul 2019	INBUILD criteria^*^	30.6% (75/245)	NR	NR
Sweeney et al. (24), Australia	Registry (*n* = 118)	NR	INBUILD criteria^*^	47.5% (56/118)	15.0	NR
Nakamura et al. (25), Japan	Clinical cohort (*n* = 110)	2010–2016	INBUILD criteria^*^	59.1% (65/110)	NR	NR
Komatsu et al. (26), Japan	Clinical cohort (*n* = 57)	Jan 2009–Dec 2015	A relative decline of ≥10% in FVC per 24 months or the relative decline in FVC of ≥5% with decline in DLco of ≥15% per 24 months	19.3% (11/57)	NR	NR
Olson et al. (27), US	Database (*n* = 2,517)	2014–2016	Pulmonologist visit frequency: ≥4 visits in 2016, or ≥3 more visits in 2016 vs. 2014	15.0% (373/2,517)	NR	NR
EU PAS Abstract (28), Europe	Database (*n* = NR)	2014–2018	Algorithm: in Phase 1, an algorithm based on codes/keywords was used to identify the crude incidence/prevalence rate of ILD, F-ILD, IPF, non-IPF F-ILD and SSc-ILD in six European countries (Belgium, Denmark, Finland, Greece, Norway, and Portugal). In Phase 2, a subset of the non-IPF F-ILD cases identified at each center were manually reviewed and extrapolated using a weighted mean percentage calculated for each country to determine the incidence/prevalence of PF-ILDs	10.4–50.0% in 2018	2.1–14.5 in 2018^†^	6.9–78.0 in 2018^†^
Nasser et al. (3), France	Database (*n* = 30,771)	Jan 2010–Dec 2017	Algorithm: ≥3 claims each for pulmonologist consultations and PFTs within 12 months; and glucocorticoid or immunosuppressive therapy; plus palliative care, or ≥3 HRCT or CT scans, or ≥1 claim for oxygen therapy, respiratory hospitalization in an ICU following an emergency visit or lung transplant	46.8% (14,413/30,771)	4.6 in 2016	19.4 in 2016
Olson et al. (29), US	Database (*n* = 35,825)	Oct 2012–Sep 2015	Algorithm: an eligible ICD-9 code for fibrosing ILD and ≥2 pulmonary function tests or ≥2 oxygen titration tests within 90 days, ≥2 HRCT or ≥3 chest CT scans within 360 days, respiratory hospitalization, palliative care, lung transplant, any use of oxygen therapy or a corticosteroid >20 mg, or new use of immunosuppressive therapy	60.6% (21,719/35,825)	32.6	70.3
Wuyts et al. (11), Europe	Expert consensus (*n* = 5,298)	2019	INBUILD criteria^*^	31.6% (1,674/5,298)	NR	NR
Olson et al. (6), Europe, US	Systematic review + expert survey (*n* = NR)	1990–2017^§^	Expert opinion and published data	13.0% (sarcoidosis ILD) to 40.0% (RA-ILD)	NR	2.2–28.0 (includes IPF)

* *In the INBUILD trial, patients were defined as progressive when ≥1 of the following criteria: relative decline of ≥10% in FVC% predicted OR relative decline ≥5– <10% in FVC% predicted with worsening respiratory symptoms and/or increasing fibrosis on chest imaging OR worsening respiratory symptoms and increasing fibrosis on chest imaging;*

† *Estimates vary by country; the lower number represents the minimum positive predictive value adjusted value and the higher number the crude maximum value;*

§ *Year of publication of included studies. CT, computed tomography; EU, European Union; F-ILD, fibrosing ILD; FVC, forced vital capacity; HRCT, high-resolution computed tomography; ICU, intensive care unit; ILD, interstitial lung disease; IPF, idiopathic pulmonary fibrosis; NR, not recorded; PAS, post-authorization study; PF, progressive fibrosing; PFTs, pulmonary function tests; RA-ILD, rheumatoid arthritis-associated ILD; SSc-ILD, systemic sclerosis-associated ILD*.

An analysis of data from the IBM® MarketScan® Research Databases of US medical and prescription commercial claims identified 21,719 non-IPF PF-ILD patients using a specifically-developed algorithm using multiple ICD-9 codes and proxies for progression for PF-ILD case identification. The estimated age- and sex-adjusted prevalence and incidence of non-IPF PF-ILD was 70.3/100,000 persons and 32.6/100,000 person-years, respectively (29).

A longitudinal retrospective cohort study carried out using the French administrative healthcare database [Système National des Données de Santé (SNDS)] identified 14,413 non-IPF PF-ILD patients with a specifically-developed algorithm using multiple ICD-10 codes and proxies for progression for case identification. The prevalence and incidence of non-IPF PF-ILD in 2016 was estimated to be 19.4/100,000 persons and 4.6/100,000 person-years, respectively (3).

A retrospective, multinational, multicenter, two-phase, hospital database study using aggregate data was conducted. Non-IPF fibrosing ILD cases identified at each center were manually reviewed and extrapolated using a weighted mean percentage calculated for each country to determine the incidence/prevalence of PF-ILDs. The extrapolated prevalence of PF-ILDs across Europe ranged between 6.9 [positive predictive value (PPV)-adjusted] and 78.0 (crude)/100,000 persons, and the incidence between 2.1 (PPV-adjusted) and 14.5 (crude)/100,000 person-years (28).

#### Proportion of ILD Patients Estimated to Develop the Progressive Phenotype

**Figure 3:**
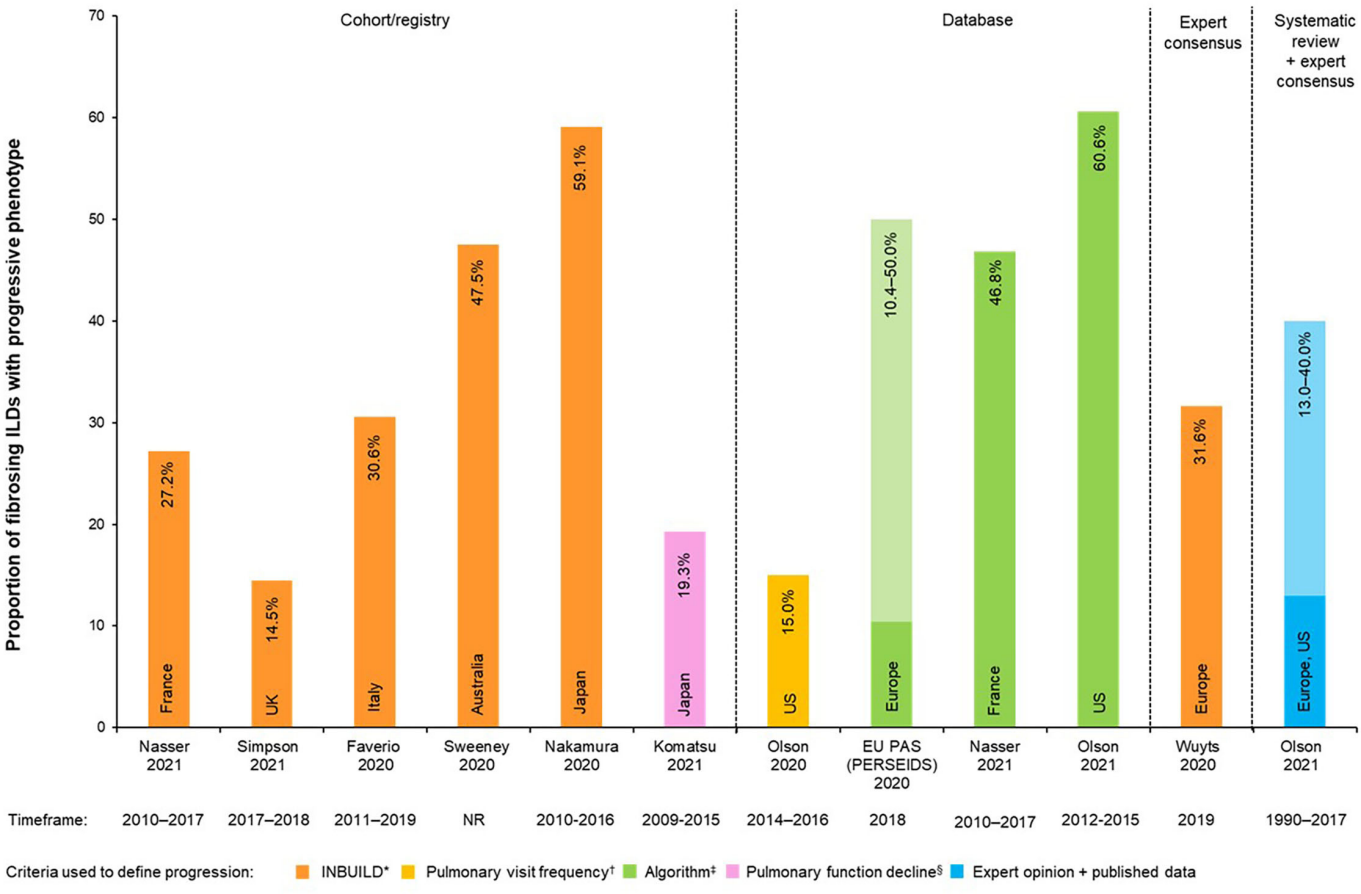
Proportions of non-IPF fibrosing ILDs with progressive phenotype (%). *In the INBUILD trial, patients were defined as progressive when ≥1 of the following criteria: Relative decline of ≥10% in FVC% predicted OR relative decline ≥5–<10% in FVC% predicted with worsening respiratory symptoms and/or increasing fibrosis on chest imaging OR worsening respiratory symptoms and increasing fibrosis on chest imaging; ^†^Pulmonology visit frequency = (≥4 visits in 2016, or ≥3 more visits in 2016 vs. 2014); ^‡^Algorithms for definition of progression were specifically designed for each study; see Table 1 for further algorithm details. ^§^Pulmonary function decline = (a relative decline of ≥10% in FVC per 24 months or the relative decline in FVC of ≥5% with decline in DLco of ≥15% per 24 months). Light vs. Dark colors on the same bar represent the percentage range (minimum to maximum proportions reported). DLco, diffusing capacity of the lungs for carbon monoxide; EU, European Union; FVC, forced vital capacity; ILD, interstitial lung disease; PAS, post-authorization study; PF-ILD, progressive fibrosing interstitial lung disease.

Twelve data sources reported on the proportion of non–IPF fibrosing ILDs estimated to develop a progressive fibrosing phenotype (Table 1, Figure 3). Up to 60.6% of patients with fibrosing ILD develop a progressive phenotype. The ranges by type of study design were: 14.5–59.1% for cohort/registry studies, 10.4–60.6% for database studies, and 13.0–40.0% for expert consensus.

One study reported on the estimated proportion of development of the progressive phenotype by different types of ILDs using a combination of published reports and an online expert survey (6): 13.0% (sarcoidosis ILD) to 40.0% (RA-ILD) of patients with ILD were estimated to develop a PF phenotype.

#### Underlying ILD Diagnoses With a Progressive Phenotype

Nine data sources reported the numbers/percentages of patients that had different underlying ILD diagnoses (Supplementary Table 2). Common underlying ILD diagnoses with a progressive fibrosing phenotype included u-ILD (17.2–71.4%), autoimmune ILDs (16.6–46.7%), chronic fibrosing HP (1.6–40.0%), other fibrosing ILDs (exposure-related ILD, sarcoidosis and other fibrosing ILD) (6.1–36.4%) and iNSIP (0.7–32.3%).

#### Survival

Eight data sources reported survival/mortality estimates for patients with PF-ILDs and show that non-IPF PF-ILD is associated with a considerable mortality risk, comparable to that of IPF (2, 22, 25, 30). Additionally, numerous studies significantly associated this increased risk of mortality with a decline in FVC (2, 21).

In a French single-center clinical cohort of 165 patients with non-IPF PF-ILD defined using INBUILD criteria, overall survival (OS) was 83% at 3 years and 72% at 5 years from ILD diagnosis date. Using multivariate Cox regression analysis, mortality was significantly associated with relative decline in forced vital capacity (FVC) ≥10% in the previous 24 months (*p* < 0.05) (21). A similar 3-year OS rate of 87.7% was shown in a retrospective Japanese database study of 87 patients with PF-ILD (including 22 cases with IPF) between January 2010 and December 2016 (25). The French SNDS database study, estimated the median OS of non-IPF PF-ILD patients from the beginning of progression (defined using proxies, see Table 1 for details) at 3.7 years (3). Survival rates were 73.7, 55.1, 42.0, and 31.6% at 1, 3, 5, and 8 years, respectively (3).

In a retrospective observational study of 1,749 non-IPF fibrosing ILD patient referrals across nine UK centers, patients with PF-ILD (*n* = 253) had a significantly higher mortality compared with those with non-PF-ILD (*n* = 1,496) [hazard ratio (HR) 3.32; 95% confidence interval (CI) 2.53–4.37; *p* ≤ 0.001] and was comparable to patients with IPF (HR 1.06; 95% CI 0.84–1.35; *p* = 0.6) (22).

A cohort study of 75 patients with non-IPF PF-ILD attending two Italian referral centers, found that disease progression (defined as a FVC decline of >5% associated with worsening respiratory symptoms or increasing extent of fibrotic changes on imaging) occurred a median of 18 months from initial ILD diagnosis (23). Progression was associated with early mortality, with patients exhibiting a median survival rate from the date of ILD progression of 3 [interquartile range (IQR) 2–5] years, and 2- and 3-year mortality rates of 4 and 20%, respectively (23).

In an Australian single-center retrospective analysis (January 2014–December 2019) patients with non-IPF PF-ILD (*n* = 267) exhibited a similar mortality risk to patients with IPF (*n* = 222) (HR 1.33; 95% CI 0.79–2.24) (30). Similarly, in a Japanese single-center retrospective study patients with non-IPF PF-ILD (*n* = 11) exhibited a significantly worse median (95% CI) OS compared with patients with non-IPF, non-PF-ILD (*n* = 46) [5.7 (3.8–not evaluable, NE) vs. 10.5 (5.0–NE) years; *p* = 0.02], and a numerically worse median (95% CI) OS compared with patients with IPF (*n* = 34) [5.7 (3.8–NE) vs. 9.1 [5.9–NE] years; *p* = 0.51] (26).

In clinical trial populations, a comparison of data from the placebo-treated non-IPF PF-ILD patients in the INBUILD trial (*n* = 324) and the placebo-treated IPF patients in the INPULSIS trials (*n* = 423), found that ~50% of patients in both the INBUILD and INPULSIS populations experienced a relative decline in FVC >10% predicted at week 52 (2). This decline was associated with an increased risk of death in the INBUILD (HR 3.64; 95% CI 1.29–10.28; *p* = 0.015) and INPULSIS (HR 3.95; 95% CI 1.87–8.33; *p* < 0.001) trials, indicating that similar to IPF, a decline in FVC is associated with an increased risk of early mortality in patients with other PF-ILDs (2).

### Humanistic Burden of Non-IPF PF-ILD

Limited data reported the humanistic burden of PF-ILD. Those studies identified showed that non-IPF PF-ILD negatively impacts both patients' and unpaid carers' quality of life (QoL), affecting their mental and physical health (9, 11, 19, 31, 32). These results are summarized below.

#### Symptoms

In the Living with Pulmonary Fibrosis (L-PF) study (*n* = 20), common symptoms reported by patients included dyspnea (95.0%), cough (95.0%), and fatigue (90.0%) (9). Similarly, patients in the King's Brief Interstitial Lung Disease (K-BILD) study (*n* = 20), commonly reported dyspnea (100.0%), chest tightness (85.0%), and chest wheezing/whistling (80.0%) (32). In the INBUILD trial, placebo-treated patients exhibited a significantly worse L-PF dyspnea score, cough score, symptoms score, impacts score, and total score compared with nintedanib-treated patients (all *p* < 0.05) (31). Furthermore, experts in the BUILDup study estimated that up to 45.6% of patients suffer fatigue (prevalence of cough and dyspnea were not reported) (11).

Dyspnea, cough and fatigue are strong drivers of HRQoL impairment in PF-ILD (9, 19). In IPF and other PF-ILDs, cough can affect sleep and willingness to participate in social activities, dyspnea can limit patients' ability to carry out physical activities, and fatigue can lead to decreased social participation, physical deconditioning, low mood and isolation (19).

#### Activities of Daily Living

Experts in the BUILDup study agreed that QoL in non-IPF PF-ILD is directly related to lung function (11). In addition, >93.0% of those surveyed agreed that non-IPF PF-ILD affects patients' QoL in emotional, social and financial domains. Experts estimate that 48.1% of patients had total permanent disability and 22.8% had lost their job because of disability (11).

In the L-PF study, 90.0% (18/20) of patients reported an impact on daily living; of these, 88.9% (16/18) reporting difficulty in completing housework, gardening, and other daily chores (9). A large proportion (16/20; 80.0%) reported physical impairments such as difficulty in walking and exercising, whilst 40.0% of patients (8/20) reported a negative impact of PF-ILD on their sleep (9).

In the K-BILD study (*n* = 20), 100.0% of patients reported that their disease interfered with their job or other daily tasks, 85.0% reported that they avoided tasks that made them short of breath, and 70.0% reported that their disease limited them in carrying things, such as groceries (32).

#### Psychological Burden

Besides posing a substantial threat to patients' physical wellbeing, PF-ILD can negatively impact patients' emotional and mental wellbeing. Depression is common, affecting 27.2% of patients with non-IPF PF-ILD, as estimated by experts in the BUILDup study (11). In the L-PF study (*n* = 20), 70.0% of patients reported negative effects of PF-ILD on their mental health, including stress and anxiety, getting frustrated easily, and feelings of fear and concern (9). In the K-BILD study (*n* = 20), 95.0% of patients reported that they worried about the seriousness of their lung symptoms, making them feel anxious (85.0%), annoyed or down (80.0%), and even suicidal (60.0%) (32).

#### Impact of PF-ILD on Caregivers

The BUILDup study assessed the impact of non-IPF PF-ILD on unpaid carers' QoL (11). A total of 85.0–90.0% of experts surveyed agreed that non-IPF PF-ILD negatively impacts unpaid carers' QoL in terms of sleep and health, daily activities, emotional wellbeing, social life, and finances. There is also a significant time burden on caregivers, with an estimated 60.5% of patients needing support from an unpaid carer such as a partner, family member, or neighbor, for 29.8 h per week (11).

#### Impact of Acute Exacerbations

AEs are the rapid deterioration of lung function, observed as a marked and recent increase in dyspnea and hypoxemia (10). They are an unpredictable serious life-threatening event and can occur at any time during the disease course (10). In the BUILDup study, experts estimated that 19.7% of patients with PF-ILD experienced an AE in the last year, compared with 7.2% of patients with non-/slow-PF-ILD (11). Similarly, experts estimated that the number of patients who experienced >1 exacerbation during the last year was >3 times higher in PF-ILD patients than in patients with non-/slow-PF-ILD (6.1 vs. 1.7%) (11).

Experts agree that AEs of PF-ILD directly impact a patient's QoL, disease progression and survival. Moreover, they reported that progression had a significant impact on QoL on both PF-ILD patients and their unpaid carers. Progression affected daily functioning and had a social and emotional impact on both groups (11).

### Economic Burden of Non-IPF PF-ILD

#### Impact of PF-ILD on Healthcare Resource Use and Direct Costs

Five studies were identified that reported on the impact of non-IPF PF-ILD on HCRU and direct costs (Table 2). These studies reported that PF-ILD is associated with significantly higher HCRU and direct costs compared with the non-progressive or slow-progressive phenotypes. In addition, resource use and costs increase over time and are higher in the period after diagnosis of progression compared to the period before. Hospitalization represented the majority of the direct costs of PF-ILD.

**Table 2:** Studies reporting the economic burden of non-IPF PF-ILD.

**References, country, currency, cost year**	**Study design, No. of non-IPF PF-ILD patients**	**Criteria used to determine progression**	**Costs included**	**HCRU, direct and indirect costs, and productivity changes reported**
Olson et al. (27), US, US$, cost year 2014–2016	Claims database (*n* = 373)	Pulmonologist visit frequency (≥4 in 2016, or ≥3 more visits in 2016 vs. 2014)	Healthcare costs	• Patients with PF-ILD had higher HCRU across all healthcare settings (physician's office, ER, hospital, and other healthcare centers) compared with non-IPF ILD population • Mean annual costs per patient US$35,364 (1.7 times higher than for non-IPF ILD) • 83.6% of mean annual costs per patient were claims associated with hospitalization
Wuyts et al. (11), Europe, €, cost year 2019^*^	Expert consensus (*n* = 1,674)	INBUILD criteria^†^	Healthcare costs	• Average total annual cost per patient €34,530 (1.8 times higher than for non-IPF ILD) • Costs per visits, hospitalizations, and tests were the main cost drivers (data not shown) • Average annual cost per patient of acute exacerbations €8,101 (3.0 times higher than for non-IPF PF-ILD • PF-ILD patients had higher resource use compared with non-IPF ILD • 26.7% of non-IPF PF-ILD patients retired early due to illness • 22.8% of non-IPF PF-ILD lost job due to disability (vs. 8.8% for non-IPF ILD) • 20.3% of non-IPF PF-ILD require a paid carer, for an average of 8 h per week • 60.5% of non-IPF PF-ILD patients require an unpaid for 29.8 h per week
Nasser et al. (3), France, €, cost year NR	Database (*n* = 14,413)	Algorithm: ≥3 claims each for pulmonologist consultations and PFTs within12 months; and glucocorticoid or immunosuppressive therapy; plus palliative care, or ≥3 HRCT or CT scans, or ≥1 claim for oxygen therapy, respiratory hospitalization in an ICU following an emergency visit or lung transplant	Healthcare costs	• 3,727 (95.2%) patients had ≥1 hospitalization during the follow-up period, with an annual median (IQR) hospitalization rate of 3.9 (1.7–9.5) per year • 89.2% of patients had pulmonary imaging, including 85.0% who had a chest X-ray and 69.2% who had a chest HRCT scan • Mean annual healthcare costs per non-IPF PF-ILD patient were €81,286 • 67.3% of these costs are attributable to hospitalizations
Singer et al. (33), US, US$, cost year 2016–2019 (adjusted to 2019)	Database (*n* = 11,025)	Algorithm: proxies used for progression NR	Healthcare costs	• The wPPPM mean ± SD number of hospitalizations was 3 × higher among the progressive cohort vs. the non-progressive cohort (0.09 ± 0.16 vs. 0.03 ± 0.09; *p* < 0.001) • Among patients with ≥1 hospitalization, the wPPPM mean ± SD number of hospitalized days was 1.6 ± 2.4 vs. 1.0 ± 1.3 for the progressive vs. non-progressive cohort, respectively; *p* < 0.001 • Total wPPPM mean ± SD costs were twice as high for the progressive cohort vs. the non-progressive cohort (US$4,382 ± 9,597 vs. US$2,243 ± 4,162, *p* < 0.001)
Olson et al. (34), US, US$, cost years variable	Database (*n* = 14,722, PF-ILD; *n* = 5,840, HCRU; *n* = 5,815, costs)	Definition of progression based upon occurrence of any proxies for progression (index) (e.g., ≥2 pulmonary function tests within 90 days, ≥2 HRCTs within 1 year or oxygen use), on or following a fibrosing ILD diagnosis	Healthcare costs	• Higher mean ± SD number of outpatient visits during 1–year follow-up (41.9 ± 30.2) than at baseline (25.7 ± 20.9) • Higher mean ± SD number of inpatient admissions was 0.7 ± 1.2) during follow-up and 0.5 ± 0.9 at baseline • Higher mean ± SD outpatient costs during follow-up (US$24,711 ± 51,429) than baseline US$17,075 ± 37,987) • Higher mean ± SD inpatient costs during follow-up (US$20,746 ± 88,880) than baseline US$14,883 ± 53,404) • Higher mean ± SD total costs during follow-up (US$54,215 ± 116,833) than at baseline (US$37,340 ± 7,423)
Birring et al. (32), US and Germany, no costs	Interview (*n* = 20)	Prespecified PF-ILD criteria (e.g., ≥10% relative decline in FVC% predicted)	None	• 100.0% (20/20) of patients reported that their disease interfered with their job or other daily tasks • 50.0% (10/20) reported that they were financially worse off as a consequence of their lung condition.

* *Average calculated from quarter 1–3 of 2019;*

† *In the INBUILD trial, patients were defined as progressive when ≥1 of the following criteria: Relative decline of ≥10% in FVC% predicted OR relative decline ≥5– <10% in FVC% predicted with worsening respiratory symptoms and/or increasing fibrosis on chest imaging OR worsening respiratory symptoms and increasing fibrosis on chest imaging. AU$, Australian dollar; CT, computed tomography; ER, emergency room; FVC, forced vital capacity; HCRU, healthcare resource use; HRCT, high-resolution computed tomography; ICU, intensive care unit; ILD, interstitial lung disease; IPF, idiopathic pulmonary fibrosis; IQR, interquartile range; NR, not recorded; PF, progressive fibrosing; PFT, pulmonary function test; SD, standard deviation; US$, United States dollar; wPPPM, weighted per patient per month*.

One study compared HCRU and costs of patient with PF-ILD compared to non-/slow-PF-ILD. The BUILDup study, a cost-analysis based on expert opinion of 40 European experts in ILD management, reported that PF-ILD patients (*n* = 1,674) had higher mean resource use than non-/slow-PF-ILD patients (*n* = 3,623) during follow-up management, including healthcare professional visits (7.0 vs. 4.7 visits/patient), laboratory tests (13.6 vs. 10.6/patient), imaging tests (19.0 vs. 14.2/patient), hospitalizations (4.4 vs. 2.6/patient), and days in hospital (5.9 vs. 3.9 days/patient). A higher proportion of patients with PF-ILD required treatment, including pharmacological treatment (93.8 vs. 78.2%), oxygen therapy (29.8 vs. 7.1%), and lung transplantation (2.8 vs. 0.4%). This translated into a mean annual cost per patient that was nearly two-fold higher (1.8) for PF-ILD patients (*n* = 1,674; €34,530) compared to patients with non-/slow-PF-ILD (*n* = 3,623; €18,746). Among follow-up costs, costs per visits, hospitalizations, and tests were the main cost drivers (data not provided). The mean annual cost per patient of AEs were 3.0 times higher in patients with PF-ILD than in patients with non-/slow-PF-ILD (€8,101 vs. €2,717) (11).

In addition, a database analysis using the Optum Research Database between January 2016 and June 2019, found that the weighted per patient per month (wPPPM) mean (±SD) number of hospitalizations was significantly higher among the PF-ILD cohort (*n* = 11,025) than the non-progressive cohort (*n* = 11,025) (0.09 ± 0.16 vs. 0.03 ± 0.09; *p* < 0.001). Among patients with ≥1 hospitalization, the wPPPM mean (±SD) number of hospitalized days was >1.5 times higher for the PF-ILD cohort than the non-progressive cohort (1.6 ± 2.4 vs. 1.0 ± 1.3; *p* < 0.001). The total (±SD) wPPPM costs were twice as high for the PF-ILD cohort (*n* = 11,025) than the non-progressive cohort (*n* = 11,025) (US$4,382 ± 9,597 vs. US$2,243 ± 4,162; *p* < 0.001) (33).

Furthermore, an analysis of US-based medical insurance and electronic health records (EHRs) of patients with non-IPF ILD (*n* = 2,517) between 2014 and 2016, found that patients with PF-ILD (*n* = 373) had higher HCRU across all healthcare settings [physician's office, emergency room (ER), hospital, and other healthcare centers] compared with the non-progressive ILD population (*n* = 2,144). Most billable claims (both ILD-related claims and “any claims”) were made in the physician's office and the hospital. In the physician's office, patients with PF-ILD made 3.0 more ILD-specific claims, and 1.9 more “any claims,” compared with the ILD group (difference in mean average for 3-year period). In the hospital setting, patients with PF-ILD made 5.0 more ILD-specific claims, and 3.5 more “any claims,” compared with the ILD group. The mean annual medical costs associated with ILD-specific claims were 1.7 times higher for patients with PF-ILD than for patients with ILD (US$35,364 vs. US$20,211). Hospitalizations accounted for the majority (83.6%) of medical costs for patients with PF-ILD (data not provided) (27).

One database study reported an increase in HCRU and costs over time from PF-ILD diagnosis. An analysis of IBM® MarketScan® claims data between October 2011 and September 2015 found that patients with PF-ILD (*n* = 58,40) had a higher (mean ± SD) number of outpatient visits (including services) during 1-year follow-up (41.9 ± 30.2) than in the period before diagnosis (baseline) (25.7 ± 20.9). The mean (±SD) number of inpatient admissions were also higher (0.7 ± 1.2 vs. 0.5 ± 0.9), including more ICU admissions and respiratory-related hospitalizations. At 1-year compared with baseline, patients with PF-ILD had higher mean (±SD) outpatient costs (US$24,711 ± 51,429 vs. US$17,075 ± 37,987), higher inpatient costs (US$20,746 ± 88,880 vs. US$14,883 ± 53,404), and higher total costs (US$54,215 ± 116,833 vs. US$37,340 ± 74,323) (34).

Finally, the French SNDS database study reported that 95.2% of PF-ILD patients had at least one hospitalization for respiratory care, with an annual median (IQR) hospitalization rate of 3.9 (1.7–9.5) per year. A total of 75.2% of patients had a hospitalization due to acute events, 11.0% were hospitalized for pulmonary hypertension and 34.3% were in an intensive care unit (ICU). The mean annual healthcare cost per patient was €81,286 (*n* = 14,413). The majority (67.3%) of these costs were attributed to hospitalizations (3).

#### Impact of PF-ILD on Indirect Costs

Two studies were identified that reported the impact of PF-ILD on indirect costs. The BUILDup study found that patients with PF-ILD suffer greater indirect costs compared with the non-progressive phenotype as a result of lost work productivity through early retirement and job losses (11). A second study, assessing the relevance of the K-BILD questionnaire in patients with non-IPF PF-ILD, reported that PF-ILD negatively affects both patients' ability to work and their financial situation (32).

Experts in the BUILDup study estimated that 71.2% of PF-LD patients (*n* = 5,298) were retired and that 26.7% retired early due to illness (data not provided). Experts also estimated that 48.1% of PF-ILD patients and 19.6% of non-/slow-PF-ILD patients (*n* = 1,674) had a total permanent disability, and that 22.8% of PF-ILD patients and 8.8% of non-/slow-PF-ILD patients had lost their job because of disability. Support from a paid carer was required by 20.3% of PF-ILD patients for an average duration of 8.0 h per week, and 60.5% of PF-ILD patients needed support from an unpaid carer such as a partner, family member, or neighbor, for 29.8 h per week (data not provided) (11). In the K-BILD study (*n* = 20), 100.0% of patients reported that their disease interfered with their job or other daily tasks, and 50.0% reported that they suffered financial hardship as a consequence of their lung condition (32).

## Discussion

Our review evaluated the epidemiological, economic and humanistic burden of PF-ILD. From the studies identified, the estimated prevalence of non-IPF PF-ILD ranged from 6.9 (Europe) to 70.3 (US)/100,000 persons and the estimated incidence ranged from 2.1 (Europe) to 32.6 (US)/100,000 person-years. Estimates for the proportions of patients with non-IPF PF-ILDs that develop a progressive fibrosing phenotype range from 10.4 to 60.6%. The variation in these epidemiological estimates is a result of factors including the variation in study designs, heterogenous referral bias between centers, definitions of progression in fibrosing ILDs applied and populations (studies were conducted across several distinct geographies and varied in underlying ILD subgroup proportions). This highlights, that despite increasing recognition of the PF phenotype among ILD specialists, there are difficulties in identifying patients with PF-ILDs other than IPF in practice due to an absence of standardized diagnostic criteria, treatment guidelines, and specialist education in the field (35).

These epidemiological estimates are higher than those reported for IPF, which throughout Europe and North America has an estimated prevalence of 1.3–42.7/100,000 persons (36, 37), and an estimated incidence of 2.8–19.0/100,000 person-years (38). Nevertheless, this difference is likely a result of the aforementioned variation in non-IPF PF-ILD study designs, definitions and populations, and the fact that the highest epidemiological estimates we identified for non-IPF PF-ILD (70.3/100,000 per persons and 32.6/100,000 person-years) are likely the upper limit of the true estimates as the study relied solely on claims data without access to confirmatory clinical information (29).

Our review confirmed that PF-ILD has a poor prognosis, characterized by early mortality. In the largest study identified (14,413 non-IPF PF-ILD patients), the estimated mean OS rate from progression was just 3.7 years (3), consistent with survival rates seen in patients with IPF (39–41). Several other real-word studies identified confirm that non-IPF PF-ILDs have a clinical course consistent with IPF, irrespective of underlying ILD diagnoses (22, 25, 30). Moreover, studies that assessed the link between FVC and survival found that mortality rate was significantly associated with a relative decline in FVC % predicted (2, 21). These results are consistent with a *post-hoc* analysis of 2,553 patients who received nintedanib or placebo in clinical trials in patients with non-IPF PF-ILD (INBUILD), IPF (TOMORROW, INPULSIS-1 and−2, and NCT01979952), and SSc-ILD (SENSCIS), and also demonstrated a strong association between decline in FVC % predicted and death (42).

Despite the limited evidence available on the humanistic burden of non-IPF PF-ILD, studies clearly demonstrated that PF-ILD has a significant impact on patients' QoL, affecting their daily lives, psychological wellbeing, careers, and personal relationships (9, 11, 32). The most prevalent symptoms of PF-ILD, dyspnea, cough, and fatigue, can limit patients' ability to carry out physical tasks and participate in social activities, leading to low mood and isolation (9, 11, 19, 32) and impact their ability to carry out daily activities, including chores, walking, and exercising (9, 32). PF-ILD also negatively impacts patients' emotional and mental wellbeing, leading to stress, anxiety, depression, and in some cases, suicidal thoughts (9, 11, 19, 32). PF-ILD affects patients' ability to look after themselves, forcing them to rely on caregivers for help (11). Caregiver burden is high, and studies in fibrosing ILDs have reported a significant emotional toll on caregivers, with feelings of helplessness, loss of independence and ability to pursue personal interests, ultimately straining personal relationships between caregivers and patients (43, 44).

Our review showed PF-ILD to be associated with significant economic burden. Several studies associated PF-ILD with significantly higher HCRU and direct costs compared with the non-progressive phenotype. PF-ILD patients required more follow-up visits (11, 27, 33), hospitalizations (11, 27, 33), days in hospital (11, 33), and laboratory/imaging tests (11). PF-ILD incurred substantial healthcare costs, with a total annual direct cost per patient of US$77,666 reported in US (27), and from €15,648 (Greece) to €81,286 (France) in Europe (3, 11). Hospitalization costs were the main cost driver (3, 11, 27), and were more frequent and longer for progressive patients. Moreover, one study reported that HCRU and direct costs for patients with PF-ILD increased over time (34), which is likely an indication of increased healthcare needs as a result of disease progression, as demonstrated in patients with IPF (45). A future challenge will be to assess if treatments that slow disease progression favorably impact the number and time to non-elective respiratory hospitalization and other direct costs.

Patients with PF-ILD experience loss of work productivity through early retirement, loss of job due to illness, and financial burden (11). As patients with non-IPF PF-ILD are typically younger than patients with IPF (29, 38, 46), it is anticipated that the burden of disease in terms of lost work productivity will be greater for non-IPF PF-ILD patients, who are more likely to be of working age and more vulnerable to productivity loss (47, 48).

Numerous studies identified in this review expressed a requirement for disease-modifying agents that reduce the significant burden experienced by patients with PF-ILD (11, 19, 21). In IPF, the emergence of antifibrotic therapies have transformed the landscape of disease management, with established efficacy in slowing disease progression, reducing AEs, and improving survival (24, 49, 50). Both antifibrotics used to treat IPF, nintedanib, and pirfenidone, have been evaluated in non-IPF PF-ILDs. Nintedanib has recently been approved for use in chronic fibrosing ILDs with a progressive phenotype other than IPF based on the results of the INBUILD trial, in which nintedanib consistently slowed progression of lung fibrosis in a broad range of non-IPF PF-ILDs (8, 22). Pirfenidone, despite demonstrating potential beneficial effects in slowing disease progression in patients with several types non-IPF PF-ILD [connective tissue disease-ILD, asbestosis, iNSIP and fibrotic HP in the RELIEF study (51), and in u-ILD (NCT03099187) (52, 53)] has yet to reach the latter stages of clinical development in non-IPF PF-ILDs (51, 52).

In addition to reducing the rate of lung function decline, evidence from the INBUILD trial suggests nintedanib may reduce the mortality and AE burden associated with PF-ILD (8). INBUILD data also indicate that nintedanib may slow worsening of dyspnea and prevent worsening of cough (31), the most salient and burdensome symptoms for patients with PF-ILD (9, 19, 32).

In IPF, there is evidence to suggest that patients who initiate nintedanib soon after IPF diagnosis may have a reduced hospitalization risk and lower medical costs (54). As QoL and survival rate are strongly associated with lung function (2, 11, 21, 42), coupled with HCRU and medical costs increasing over time for non-IPF PF-ILD patients (34), early nintedanib treatment may theoretically reduce the substantial humanistic, economic and survival burden associated with PF-ILD.

There is a need for further research to quantify the epidemiological, humanistic, and indirect cost burden of PF-ILD, using robust, well-designed methods to improve the validity and reliability of results. In particular, future epidemiological studies would benefit from a rigorous approach to study design using validated algorithms, to further substantiate the broad epidemiological estimates we identified for PF-ILD. Currently, there are also an absence of dedicated PROMs and PREMs in this field (9). Whilst tools such as the L-PF have demonstrated applicability and sensitivity to change in PF-ILD in the context of the INBUILD trial, and are expected to be used to measure HRQoL in PF-ILD in future (9, 32), current tools have their limitations in measuring preservation of QoL in this chronic disease (9, 55) Consequently, further work is needed to develop PROMs/PREMs specific to this disease.

Broad search terms and rigorous selection and screening methodologies were undertaken to perform this structured literature review, resulting in a comprehensive review of the currently available literature on the burden of non-IPF PF-ILD. A limitation of this study is that as PF-ILD has only recently been defined as a disease phenotype (5), data on the burden of PF-ILD were limited. In addition, because PF-ILD is a newly recognized disease phenotype without an available ICD-10 code outside of the US (6, 12), there were heterogenous definitions of progression used to identify PF-ILD patients across studies, contributing to the varied epidemiological estimates identified. Furthermore, PF-ILD is progressive despite appropriate management for the underlying disease, which often includes immunomodulation; however, information about such treatment is not available from the literature reviewed here. Finally, the cost data presented in this review are as reported (without being inflated to current prices and converted to single currency) as cross-country comparisons are hindered by differences in healthcare systems, cost component data, and methodologies across the included studies.

## Conclusion

This review highlights the need for further high-quality research in the field. Nonetheless, the data indicate that PF-ILD places a considerable humanistic burden on both patients and caregivers, and a substantial economic burden on healthcare systems, patients, and society. There is a clear need for the early treatment of patients with PF-ILD with disease-modifying therapies such as antifibrotics, to ameliorate the burden of this progressive disease.

## Author Contributions

RT, LN, and SL of Maverex Limited provided writing, editorial support, and formatting assistance, which was contracted and funded by BI. VC and MB wrote sections of the manuscript. All authors contributed to the article and approved the submitted version.

## Funding

This work was supported by Boehringer Ingelheim International GmbH.

## Conflict of Interest

This study received funding from Boehringer Ingelheim International GmbH. The funder was involved in interpretation of data and the decision to submit it for publication. The funder had no role in the study design, collection, analysis, or the writing of this article. VC reports personal fees and non-financial support from Actelion, grants, personal fees and non-financial support from Boehringer Ingelheim, personal fees from Bayer/MSD, personal fees and non-financial support from Roche/Promedior, personal fees from Sanofi, personal fees from Celgene/BMS, personal fees from Galapagos, personal fees from Galecto, personal fees from Shionogi, personal fees from Astra Zeneca, personal fees from Fibrogen, personal fees from RedX, personal fees from PureTech, outside the submitted work. RT, LN, and SL report consultancy fees from Boehringer Ingelheim, during the conduct of the study. MB reports being an employee of Boehringer Ingelheim. RT, LN, and SL were employed by Maverex Limited.

## Publisher's Note

All claims expressed in this article are solely those of the authors and do not necessarily represent those of their affiliated organizations, or those of the publisher, the editors and the reviewers. Any product that may be evaluated in this article, or claim that may be made by its manufacturer, is not guaranteed or endorsed by the publisher.
