# Supplementary material for: The Burden of Progressive-Fibrosing Interstitial Lung Diseases
Source: Front Med (Lausanne). 2022 Feb 1;9:799912. doi: 10.3389/fmed.2022.799912 (PMC8843847; doi:10.3389/fmed.2022.799912)
Supplement: Supplementary file 1 [file Data_Sheet_1.docx]

**Supplementary materials**

Supplementary Table S1 Search strategy

| Ovid MEDLINE(R) and Epub Ahead of Print, In-Process, In-Data-Review & Other Non-Indexed Citations and Daily <1946 to June 16, 2021> | |
| --- | --- |
| Population | (PF‑ILD or PFILD or ((progressi* or advanced) adj5 (fibros* interstit* adj (lung* or pulmon*)))).mp. OR Lung Diseases, Interstitial/ OR ILD.tw. OR Pulmonary Fibrosis/ OR (interstitial$ adj3 (lung$ adj3 disease$)).mp. OR (interstitial$ adj3 (fibros$ or pneumonitis or pneumonia or pneumopathy)).mp. OR alveolitis.mp. OR (diffuse* adj3 parenchymal*).mp. OR Bronchiolitis Obliterans/ OR (bronchiolitis adj obliterans).mp. OR (pneumoconiosis or pneumokoniosis or pneumonoconiosis).mp. OR Pneumoconiosis/ OR bagassosis.mp. OR ((bird$ or farmer$ or pigeon$ or avian$ or budgerigar$) adj (lung$ or disease$)).mp. OR Bird Fancier's Lung/ OR (asbestosis or byssinosis or siderosis or silicosis or berylliosis or anthracosilicosis or silicotuberculosis).mp. OR ((pulmonary$ or lung$) adj3 reticulation$).mp. OR ((pulmonary$ or lung$) adj3 fibros$).mp. OR Alveolitis, Extrinsic Allergic/ OR (connective adj3 lung$).mp. OR (allerg$ adj3 pneumonitis).mp. OR Cryptogenic Organizing Pneumonia/ OR cryptogenic organi#ing pneumonia.mp. OR Idiopathic Interstitial Pneumonias/ OR IIP.tw. OR (hypersensitivity adj3 pneumonia$).mp. OR pleuroparenchymal fibroelastosis.mp. AND (progressive or progressing or progression).tw,kf,hw. |
| Epidemiology | (incidence or prevalence or prediction or prognosis).ti,kf,hw. OR ((predict* or prognos*) adj3 (factor? or indicat* or model*)).ti,ab,kf,kw. OR (risk* adj3 (factor? or indicat* or model*)).ti,ab,kf,kw. OR mortality/ or death/ or "cause of death"/ or survival rate/ OR (survival or morbidit* or comorbid* or co-morbid*).ti,kf,hw. OR (death? or mortality or fatal*).ti,kf. OR ((death? or mortalit* or fatal*) adj3 (cause? or causal or compar* or ratio or rate? or register* or registries or statistic*)).ti,ab,kf,kw. OR life table?.mp. |
| Healthcare resource use and costs | Economics/ OR “Costs and Cost Analysis"/ OR Economics, Dental/ OR exp Economics, Hospital/ OR Economics, Medical/ OR Economics, Nursing/ OR Economics, Pharmaceutical/ OR exp "Fees and Charges"/ OR exp Budgets/ OR (budget$ or financ$).tw. OR (economic* or cost or costs or costly or costing or price or prices or pricing or pharmacoeconomic* or pharmaco-economic* or expenditure or expenditures or expense or expenses or financial or finance or finances or financed).kf,ti. OR (economic* or cost or costs or costly or costing or price or prices or pricing or pharmacoeconomic* or pharmaco-economic* or expenditure or expenditures or expense or expenses or financial or finance or finances or financed).ab. /freq=2 OR exp Health Care Costs/ OR exp Drug Costs/ OR exp Hospitalization/ OR exp "Cost of Illness"/ OR Health Expenditures/ OR exp Drug Utilization/ OR exp "Utilization Review"/ OR exp Cost-Benefit Analysis/ OR (cost? adj2 (illness or disease or sickness or health care or healthcare or treatment or direct or indirect or medical or resource)).tw. OR (burden? adj2 (illness or disease? or condition? or economic*)).tw. OR "global burden of disease"/ OR (utili?ation adj2 (health or medical or resource)).tw. OR (out-of-pocket adj2 (payment? or expenditure? or cost? or spending or expense?)).tw. OR (expenditure? adj3 (health or direct or indirect)).tw. OR (expenditure? not energy).tw. OR (health care cost$ or hospitali?ation or health care utili?ation or bed day$ or cost of illness).tw. OR (value adj2 (money or monetary)).tw. OR (cost* adj2 (effective* or utilit* or benefit* or analy* or outcome or outcomes)).tw. OR (expenditure or value for money or budget).tw. |
| Health-related quality of life | quality-adjusted life years/ (13017) OR (qaly$ or qald$ or qale$ or qtime$).tw. OR (quality adjusted or adjusted life year$ or quality adjusted life year$).tw. OR (disability adjusted life or daly$).tw. OR ((index and wellbeing) or (quality and wellbeing) or qwb or qwbsa).tw. OR (multiattribut$ or multi attribut$).tw. OR (utilit$ adj2 (value$ or cost$ or health or analys$ or index or indices)).tw. OR disutilit$.tw. OR (hsuv or hsuvs).tw. OR (health$1 year$1 equivalent$1 or hye$).tw. OR (illness state$ or health state$ or health status$).tw. OR (euro qual or euro qual5d or euro qol5d or eq-5d or eq5-d or eq5d or euroqual or euroqol or euroqual5d or euroqol5d).tw. OR (hui or hui1 or hui2 or hui3 or hui-1 or hui-2 or hui-3).tw. OR health utilit$.tw. OR quality of wellbeing$.tw. OR (quality of well being or index of wellbeing or index of well being or qwb).tw. OR (short form$ or short-form$ or shortform$).tw. OR (sf36$ or sf-36$ or sf 36 or sf6 or sf 6 or sf-6 or sf6d or sf 6d or sf-6d or sf8 or sf-8 or sf 8 or sf12 or sf-12 or sf 12 or sf16 or sf-16 or sf 16 or sf20 or sf-20 or sf 20 or sf thirtysix or sf thirty six).tw. OR (15d or 15-d or 15 dimension).tw. OR (standard gamble$ or sg).tw. OR (time trade off$1 or time tradeoff$1 or tto or timetradeoff$1).tw. OR (visual analog$ scale$ or EQ-VAS).tw. OR discrete choice experiment$.tw. OR (patient? adj3 report* adj3 (outcome? or experience?)).tw. |
| Exclusions | Non-English language, animals not humans, case report, editorials |

**For information only**

Supplementary Table S2 Underlying ILD diagnoses with a progressive phenotype

|  | **Flaherty 2019** [8]  **(=663), % (n)** | **Nasser 2021** [26] **(n=165), % (n)** | **Olson 2021** [21]  **(n=21719), % (n)** | **Nasser 2021** [3] **(n=14413), % (n)** | **Simpson 2021**[25] **(=253), % (n)** | **Faverio 2020** [27] **(n=75), % (n)** | **Komatsu 2021**[28]**, (n=11), % (n)** | **Sweeney 2020** [50] **(n=56), % (n)** | **Nakamura 2020** [23] **(n=65), % n** |
| --- | --- | --- | --- | --- | --- | --- | --- | --- | --- |
| **Criteria used to determine progression** | **INBUILD criteria*** | **INBUILD criteria*** | **Algorithm^†^** | **Algorithm^†^** | **INBUILD criteria*** | **INBUILD criteria*** | **Pulmonary function decline^‡^** | **INBUILD criteria*** | **INBUILD criteria*** |
| Chronic fibrosing hypersensitivity pneumonitis | 26.1% (173) | 8.5% (14) | 1.6% (346) | 5.1% (728) | 33.2% (84) | 17.3% (13) | – | 25.0% (14) | 40.0% (26) |
| Idiopathic non-specific interstitial pneumonia | 18.9% (125) | 7.3% (12) | 0.7% (150) | 21.6% (3113) | 14.2% (36) | 28.0% (21) | 18.2% (2) | – | 32.3% (21) |
| Unclassifiable idiopathic interstitial pneumonia | 17.2% (114) | 31.5% (52) | 71.4% (15515) | – | 17.3% (44) | – | 18.2% (2) | 28.6% (16) | – |
| Interstitial pneumonitis with autoimmune features | – | 1.2% (2) | – | – | – | – | – | – | – |
| Autoimmune ILD | 25.6% (170) | 46.7% (77) | – | – | 16.6% (42) | – | 27.3% (3) | – | – |
| Rheumatoid arthritis-ILD | 13.4% (89) | 4.2% (7) | 8.2% (1777) | 17.5% (2521) | – | – | 0% (0) | – | – |
| Systemic sclerosis-ILD | 5.9% (39) | 26.1% (43) | 2.8% (618) | 6.3% (907) | – | – | 18.2% (2) | – | – |
| Dermatomyositis-ILD 12 | – | 7.3% (12) | – | – | – | – | 0% (0) | – | – |
| Mixed connective tissue disease-ILD | 2.9% (19) | 6.1% (10) | 0.3% (62) | 4.5% (655) | – | 20.0% (15) | – | 21.4% (12) | 10.8% (7) |
| Other autoimmune-ILD (Sjogren's syndrome, systemic lupus erythematosus and other autoimmune ILD) | 3.5% (23) | 10.3% (17) | – | 10.4% (1503) | – | – | 9.1% (1) | – | – |
| Other fibrosing ILDs (exposure-related ILD, sarcoidosis and other fibrosing ILD) | 12.2% (81) | 6.1% (10) | 7.6% (1,641) | 24.2% (3486) | 18.6% (47) | 29.3% (22) | 36.4% (4) | – | 16.9% (11) |

*In the INBUILD trial, patients were defined as progressive when ≥1 of the following criteria: Relative decline of ≥10% in FVC% predicted OR relative decline ≥5% to <10% in FVC% predicted with worsening respiratory symptoms and/or increasing fibrosis on chest imaging OR worsening respiratory symptoms and increasing fibrosis on chest imaging; ^†^Algorithms for definition of progression were specifically designed for each study; see Table 1 in main paper for further algorithm details. **^‡^**Pulmonary function decline = a relative decline of ≥10% in FVC per 24 months or the relative decline in FVC of ≥5% with decline in DLco of ≥15% per 24 month). DLco, diffusing capacity of the lungs for carbon monoxide FVC, forced vital capacity; ILD, interstitial lung disease; NR, not recorded
